# Removal of Dinotefuran, Thiacloprid, and Imidaclothiz Neonicotinoids in Water Using a Novel *Pseudomonas monteilii* FC02–Duckweed (*Lemna aequinoctialis*) Partnership

**DOI:** 10.3389/fmicb.2022.906026

**Published:** 2022-06-09

**Authors:** Xiao-Yu Cai, Man Xu, Yu-Xuan Zhu, Ying Shi, Hong-Wei Wang

**Affiliations:** ^1^Nanjing Institute of Environmental Science, Ministry of Ecology and Environment of China, Nanjing, China; ^2^Key Laboratory of Pesticide Environmental Assessment and Pollution Control, Ministry of Ecology and Environment of China, Nanjing, China

**Keywords:** plant growth-promoting bacteria, duckweed, neonicotinoid, plant–bacteria interaction, bioremediation

## Abstract

Neonicotinoids (NEOs) are the most widely used insecticides in the world and pose a serious threat to aquatic ecosystems. The combined use of free-floating aquatic plants and associated microorganisms has a tremendous potential for remediating water contaminated by pesticides. The aim of this study was to determine whether plant growth-promoting bacteria (PGPB) could enhance the phytoremediation efficiency of duckweed (*Lemna aequinoctialis*) in NEO-contaminated water. A total of 18 different bacteria were isolated from pesticide-stressed agricultural soil. One of the isolates, *Pseudomonas monteilii* FC02, exhibited an excellent ability to promote duckweed growth and was selected for the NEO removal experiment. The influence of strain FC02 inoculation on the accumulation of three typical NEOs (dinotefuran, thiacloprid, and imidaclothiz) in plant tissues, the removal efficiency in water, and plant growth parameters were evaluated during the 14-day experimental period. The results showed that strain FC02 inoculation significantly (*p* < 0.05) increased plant biomass production and NEO accumulation in plant tissues. The maximum NEO removal efficiencies were observed in the inoculated duckweed treatment after 14 days, with 92.23, 87.75, and 96.42% for dinotefuran, thiacloprid, and imidaclothiz, respectively. This study offers a novel view on the bioremediation of NEOs in aquatic environments by a PGPB–duckweed partnership.

## Introduction

Neonicotinoids (NEOs) are the most widely used class of insecticides worldwide (Pang et al., 2020; Schmidt et al., 2022; Zhang and Lu, 2022). They are commonly used for the protection of crops (e.g., grain, oilseed, vegetables, and fruit) against a range of pests (Liu et al., 2021). However, an increasing number of studies have reported that NEOs may be harmful to non-target organisms including aquatic organisms, pollinators (e.g., honeybees), and vertebrate wildlife (Pandey et al., 2009; Pang et al., 2020; Schmidt et al., 2022). Due to their wide usage, relatively long half-life in soil, low soil adsorption, and high solubility in water, NEOs have been reported in surface waters and groundwater adjacent to agricultural areas (Anderson et al., 2015; Mahai et al., 2021; Zhang and Lu, 2022). In some studies, NEOs have been detected in natural waters at or above concentrations of acute and chronic exposure thresholds for many aquatic invertebrate species (Van Dijk et al., 2013; Anderson et al., 2015; Schmidt et al., 2022).

Recently, various approaches have been applied to remove pesticides from aqueous environments, including electrochemical oxidation, photocatalysis, Fenton processes, and membrane separation (Malakootian et al., 2020; Zhang et al., 2020). Compared with the high cost and increased possibility of secondary pollution of these methods, phytoremediation is considered an effective method for removing pesticides in aquatic environments (Ekperusi et al., 2019; Liu et al., 2021). Plants remove organic pollutants through several biologically active processes, such as accumulation, transformation, stabilization, and mineralization (Hu et al., 2019; Ishizawa et al., 2020). Lemnaceae (commonly known as duckweeds) are free-floating aquatic plants distributed across the world (Gatidou et al., 2017; Ekperusi et al., 2019). Due to their fast growth rate, high adaptability to the aquatic environment, and tolerate to a high level of contaminants, duckweeds have been applied with success for the removal of organic pollutants such as polycyclic aromatic hydrocarbons, pesticides, petroleum hydrocarbons, and antibiotics (Yamaga et al., 2010; Ekperusi et al., 2019; Hu et al., 2021).

In agriculture, the inoculation of plant growth-promoting bacteria (PGPB) has been intensively researched as a promising technology to increase crop production (Lobo et al., 2019; Zhang et al., 2019). PGPB can improve plant growth through several mechanisms, such as increased nutrient uptake, production of 1-aminocyclopropane-1-carboxylate (ACC) deaminase, phytohormones and siderophores, and nitrogen fixation (Yamakawa et al., 2018; Lobo et al., 2019; Khairina et al., 2021). In recent years, phytoremediation strategy involving PGPB has been proposed as an alternative for pollutant removal in water (Rehman et al., 2018; Yan et al., 2022). Some studies have attempted to introduce PGPB to duckweed and have reported enhanced biomass production and phytoremediation efficiency of contaminants in water (Yamaga et al., 2010; Ishizawa et al., 2020). Notably, the PGPB *Acinetobacter calcoaceticus* P23-inoculated duckweed *L. minor* accelerated biomass production by 1.9–2.3-fold compared to uninoculated duckweed in a secondary sewage effluent and displayed improved nitrogen and phosphorus removal (Ishizawa et al., 2020). Moreover, PGPB-assisted phytoremediation technology of phenol can provide better duckweed growth and increase the phenol degradation rate compared with using plants alone (Yamaga et al., 2010). The importance of PGPB–plant partnerships in the remediation of organic pollutants has been confirmed in different studies (Rani et al., 2019; Ishizawa et al., 2020; Pang et al., 2020). However, few reports have been published regarding the remediation of NEOs using a PGPB–duckweed partnership.

In this study, we first aimed to obtain a series of new PGPB for the common duckweed *Lemna aequinoctialis*. Second, we determined the effects of the inoculation of the PGPB strain on the growth and pesticide removal of *L. aequinoctialis* in three typical NEO (dinotefuran, thiacloprid, and imidaclothiz)-contaminated water.

## Materials and Methods

### Chemicals and Plants

Standards and chemicals of dinotefuran, thiacloprid, and imidaclothiz were purchased from Sigma-Aldrich (United States). The chemical structures of the three NEOs are displayed in Table 1. Methanol (HPLC grade) was purchased from Merck (Germany). The remaining reagents, which were at least of analytical grade, were purchased from Aladdin Reagent (China).

**Table 1:** Information and HPLC-MS/MS parameters for dinotefuran, thiacloprid, and imidaclothiz.

Compound	Molecular formula	Molecular structure	Retention time (min)	ESI mode	Precursor ion [M + H]^+^ (m/z)	Product ion (m/z)	Collison energy (eV)
Dinotefuran	C_7_H_14_N_4_O_3_	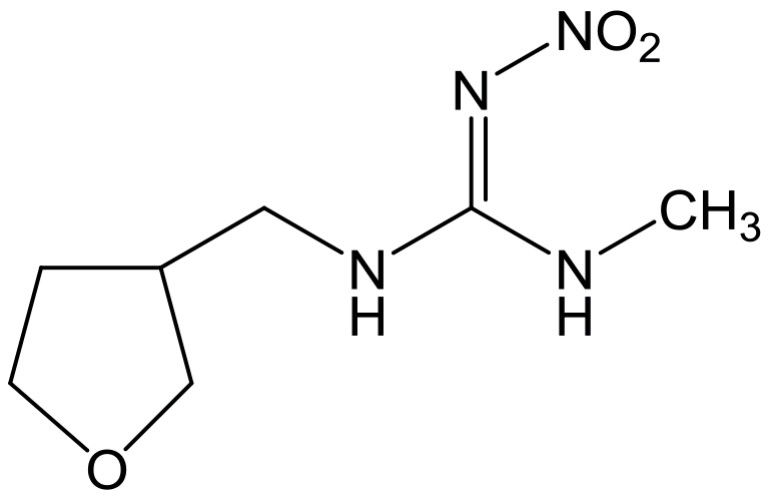	0.77	Positive	203.1	156.9, 129.1^*^, 113.1	15
Thiacloprid	C_10_H_9_ClN_4_S	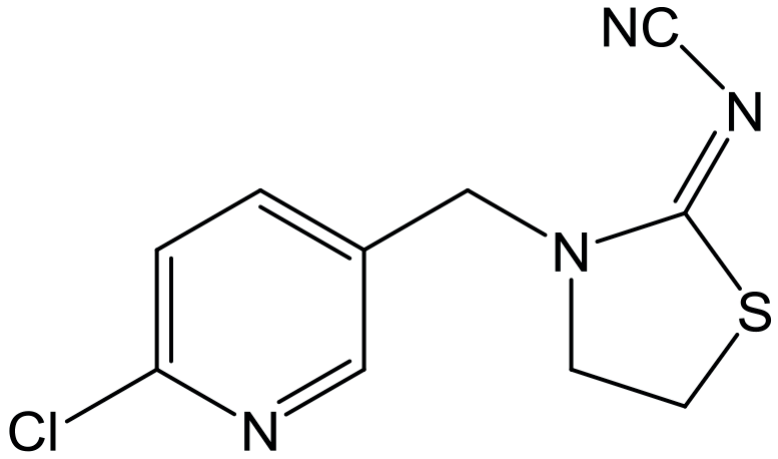	3.42	Positive	252.9	126.1^*^, 185.9	24
Imidaclothiz	C_7_H_8_ClN_5_O_2_S	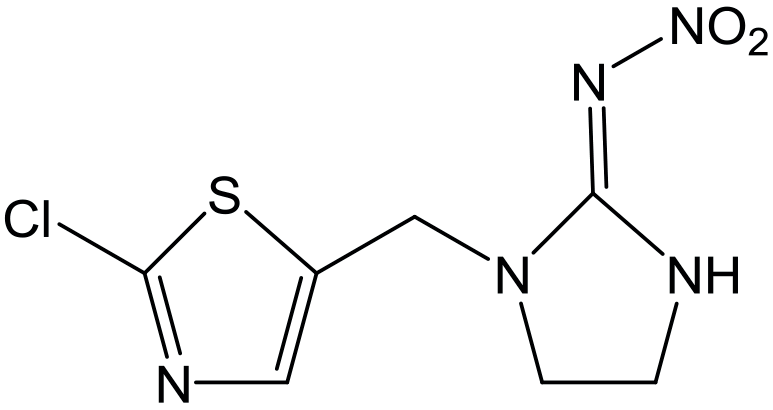	2.48	Positive	262.0	180.9^*^, 121.8, 131.7	30

* Product ion was used for quantification.

Common duckweed (*L. aequinoctialis* DKLe0261)^1^ was obtained from the China Culture Collection of Aquatic Plants (Institute of Hydrobiology, Chinese Academy of Sciences, Wuhan, China). *L. aequinoctialis* was surface-sterilized by soaking in 75% ethanol for 3 min followed by treatment with 0.5% sodium hypochlorite for 5 min. Plant sterility was ascertained by incubating the plant tissues on Luria Bertani (LB) agar (yeast extract, 5 g L^−1^; tryptone, 10 g L^−1^; NaCl, 10 g L^−1^) at 30°C for 48 h and checking for the absence of bacterial contamination. The sterilized plants were successively cultured in flasks containing sterile Hoagland medium in a growth chamber (8,000 lux; 16:8 h light–dark cycle) at 24°C. Hoagland medium contained 293 mg L^−1^ K_2_SO_4_, 36.1 mg L^−1^ KNO_3_, 103 mg L^−1^ MgSO_4_·7H_2_O, 147 mg L^−1^ CaCl_2_·2 H_2_O, 5.03 mg L^−1^ NaH_2_PO_4_·2H_2_O, 0.95 mg L^−1^ H_3_BO_3_, 3.33 mg L^−1^ FeSO_4_·7H_2_O, 0.08 mg L^−1^ ZnSO_4_·7 H_2_O, 0.39 mg L^−1^ MnCl_2_·4H_2_O, 0.03 mg L^−1^ CuSO_4_·5H_2_O, 0.39 mg L^−1^ MnCl_2_·4 H_2_O, and 0.23 mg L^−1^ H_2_MoO_4_.

### Isolation and Identification of Plant Growth-Promoting Bacteria

The soil samples for PGPB isolation were randomly collected from vegetable greenhouse, which was regularly sprayed with neonicotinoid pesticides in Nanjing Institute of Vegetable Science (118°46.61′ E, 31°43.19′ N), Nanjing, Jiangsu Province, China. The soil at this site has been defined as yellow-brown earth according to the Chinese soil classification. The collected soil (6.02 pH, 10.25 g kg^−1^ TOC, 1.75 g kg^−1^ TN, 0.023 g kg^−1^ DOC, 1.81 g kg^−1^ TP, and 20.32 g kg^−1^ TK) was air-dried, sieved (2 mm), and stored at 4°C until use. The screening method was described by Bal et al. (2013). Briefly, 1 g of soil was added to 50 ml of sterile DF salt minimal medium (Dworkin and Foster, 1958) containing 3 mM 1-aminocyclopropane-1-carboxylic acid (ACC) as the nitrogen source and incubated on an orbital shaker (30°C, 200 rpm) for 24 h. Fourfold dilutions of this culture were spread on solid DF salt minimal medium (2% agar) containing ACC (500 μM ml^−1^) and incubated for 48 h at 30°C. Bacterial colonies of different morphologies were chosen and purified. The isolated bacteria were identified by 16S rRNA gene sequencing using the universal primers 27F (5′-AGAGTTTGATCCTGGCTCAG-3′) and 1492R (5′-TACGGTTACCTTGTTACGACTT-3′). The 16S rRNA gene sequence was aligned with the sequences in GenBank database using the BLAST program. The phylogenetic tree was constructed in MEGA 6.0 by using the neighbor-joining method. The sequences were deposited in GenBank, and accession numbers were obtained.

### Effect of Isolated Bacterial Strains on *Lemna aequinoctialis* Growth

To cultivate the bacterial isolates used in the experiments, a single colony of each strain was transferred to 50 ml of liquid LB medium in an Erlenmeyer flask (250 ml) and incubated at 30°C and 200 rpm for 24 h. The bacterial cells were harvested by centrifugation (8,000 × *g*, 5 min), washed twice with sterile distilled water, and then resuspended in sterilized Hoagland medium (121°C for 20 min) with cells at an optical density at OD_600_ = 0.1. To allow bacterial isolates to attach to the plants, the surface-sterilized *L. aequinoctialis* were placed on each bacterial suspension for 24 h. The same amount of Hoagland medium without the introduction of bacterial isolates was used as a control. Then, 12 healthy duckweed fronds were transferred to a sterilized 6-well plate containing 5 ml of sterilized Hoagland medium. After 7 days of cultivation, the duckweed fresh weight was measured.

The effects on plant growth (*EPG*) are calculated using the following formula:


EPG(%)=(Wt−Wc)/Wc×100

where *Wt* is the fresh weight of bacteria-treated *L. aequinoctialis* on day 7 and *Wc* is that of the control plant.

### Plant Growth-Promoting Traits of the Selected Isolate

IAA (indole-3-acetic acid) production was tested by inoculating the strain into 100 ml of LB broth amended with 0.05% (w/v) L-tryptophan for 48 h in the dark at 30°C (Patten and Glick, 2002). IAA production was measured by a spectrophotometer at 595 nm using the Salkowski reagent (0.05 mol/l FeCl_3_ in 35% HClO_4_). The ability to solubilize insoluble phosphate was tested on Pikovaskaya’s agar medium containing 2% tricalcium phosphate (Kucey, 1987). The presence of a clear zone around the bacterial colonies after incubation for 7 d at 28°C confirmed the solubilization of phosphate. Bacterial siderophore production was determined using the method of Schwyn and Neilands (1987). The appearance of the orange-halo zone on Chrome Azural S (CAS) agar plates (28°C) after 3 d was considered positive for siderophore production. Nitrogen fixation ability was tested on nitrogen-free Ashby medium according to the process described by Kızılkaya (2008). The cell morphology of the selected isolate was detected by scanning electron microscope (SEM) imaging according to the method by Dal Cortivo et al. (2017).

### Toxicity Experiments

The surface-sterilized *L. aequinoctialis* were initially grown for 4 weeks in Hoagland medium under the conditions described by OECD Guideline 221 (OECD, 2006). Toxicity experiments were conducted in the presence of 10, 100, and 1,000 μg L^−1^ NEO compounds based on already reported environmental concentrations and on worst-case scenarios of contamination. A Hoagland medium setup without NEOs was also used as an experimental control. All toxicity experiments were performed in triplicate in 500-ml glass beakers containing 100 ml of Hoagland medium with 12 healthy fronds of *L. aequinoctialis* per petri beaker. The toxicity experiments were conducted in a growth chamber (photoperiod 14 h light; temperature 24°C; light intensity, ~8,000 lx). The duration of the experiment was 7 days, and at the end, the duckweed plant biomass (fresh weight) in each replicate was recorded.

### Removal of NEOs Using *Pseudomonas monteilii* FC02 and *Lemna aequinoctialis*

One bacterial strain (*P. monteilii* FC02) with the strongest duckweed growth-promoting ability was used in the NEO removal experiment. Treatments consisted of (1) the control (CK, no duckweed and FC02), (2) uninoculated duckweed (DW−), (3) strain FC02 alone (FC02), and (4) duckweed inoculated with strain FC02 (DW+). The chosen concentrations of NEOs (100 μg/l for each pesticide) were generally greater than those found in natural ecosystems, such as rivers and lakes, to ensure sufficient uptake for detection and measurement. In the DW− and DW+ treatments, 0.3 g fresh weight of the inoculated or uninoculated duckweed prepared as described above was transferred into a 500-ml beaker containing 100 ml of sterilized Hoagland medium. The assay started with plants covering 60 ~ 80% of the vessel area as described by Hu et al. (2019, 2021). For the FC02 treatment, 6.8 × 10^6^ cells (equivalent to the number of FC02 cells adhered to 0.3 g of duckweed as counted by an assay of colony-forming units (CFU) at 48 h) were added to 100 ml of sterilized Hoagland medium. Three pesticide stock solutions (100 mg L^−1^ for each pesticide) were added to Hoagland medium at a concentration of 100 μg L^−1^, respectively. The experiments were conducted in a growth chamber (photoperiod 14 h light; temperature 24°C; light intensity, ~8,000 lx). The removal experiments were undertaken in triplicate, and the loss of water due to evaporation was compensated by adding sterilized ultrapure water every day. In total, 144 beakers (4 treatments × 3 pesticides × 3 repeats × 4 sampling dates) were prepared to permit destructive sampling after 0, 3, 7, and 14 days. After destructive sampling, the water and plant samples were collected for the determination of the indicators described below.

### Plant Biomass and Survival of Inoculated Strain FC02

All duckweed plants were blotted using sterilized filter paper and weighed. The survival of strain FC02 in water (FC02 treatment) or adhering to duckweed (DW+ treatment) was monitored as described earlier (Khairina et al., 2021). Tenfold serial dilutions of water samples were plated in triplicate on LB-agar plates at 30°C for 48 h. The 0.1 g (fresh weight) of duckweed samples was transferred into 1.5-ml plastic tubes containing 1 ml of sterilized water and homogenized. The homogenized samples were diluted, spread onto LB agar plates, and incubated at 30°C for 48 h. The number of colony-forming units (CFUs) was counted.

### NEO Analysis in Water and Plant Samples

#### Extraction and Cleanup

Water samples (2 ml) were filtered through a 0.22-μm nylon filter and stored at −20°C before LC–MS/MS (liquid chromatography coupled to a triple quadrupole mass spectrometer) analysis. The method for extracting NEO residues in duckweed plants was a modified procedure of Muerdter and LeFevre (2019). Plant samples were dried with a vacuum freeze-drying machine. Approximately 0.2 g of each freeze-dried plant sample was ground into a fine powder with a tissue grinder. Approximately 0.1 g of homogenized sample was extracted twice with 10 ml of methanol with vortexing for 5 min and centrifugation at 8000 rpm for 5 min. The supernatants were combined and dried through a gentle nitrogen flow. Finally, the residue was dissolved in 1.0 ml of methanol and then filtered through a 0.22-μm nylon filter before LC–MS/MS analysis.

#### Chromatographic Conditions

An Agilent 1,290 Infinity LC system with an AB SCIEX Triple Quad 4,500 MS system, operated in positive and negative electrospray ionization modes, was used for the analysis of the three NEOs (dinotefuran, thiacloprid, and imidaclothiz). NEOs were separated using an Agilent Eclipse Plus C18 column (2.1 × 150 mm, 3.5 μm I.D., 0.5 ml min^−1^ flow rate, 10 min run time, 10 μl injections) maintained at 30°C. The mobile phase consisted of ultra-pure water containing 0.15% formic acid (A) and methanol (B). The percentage of A was changed linearly as follows: 90% at 0 min; 85% at 2.5 min; 70% at 5 min; and 90% at 10 min. NEOs were identified by retention time and using two or three ion products from the corresponding precursor ion. The most intense ion product was selected for quantification. Detection was performed in positive ion mode. Calibration standards (0.1–100 μg L^−1^) for each target compound were made in nutrient solution with methanol. A linear relationship was observed for all compounds (*R*^2^ > 0.999). Details of the LC–MS/MS conditions for NEOs are shown in Table 1.

### Data Analysis

All statistical analyses were performed using SPSS 22.0 (SPSS Inc., United States).

The removal efficiency of NEO is calculated by the following formula:


$$\mathrm{Removal efficiency}\;\left(\%\right)=\left[\left(C_{0}-C_{t}\right)/C_{0}\right]\times 100.$$

where C_0_ is the initial NEO concentration in the medium and C_t_ is the concentration of NEO measured at time “t” (day).

## Results and Discussion

### Isolation and Identification

**Figure 1:**
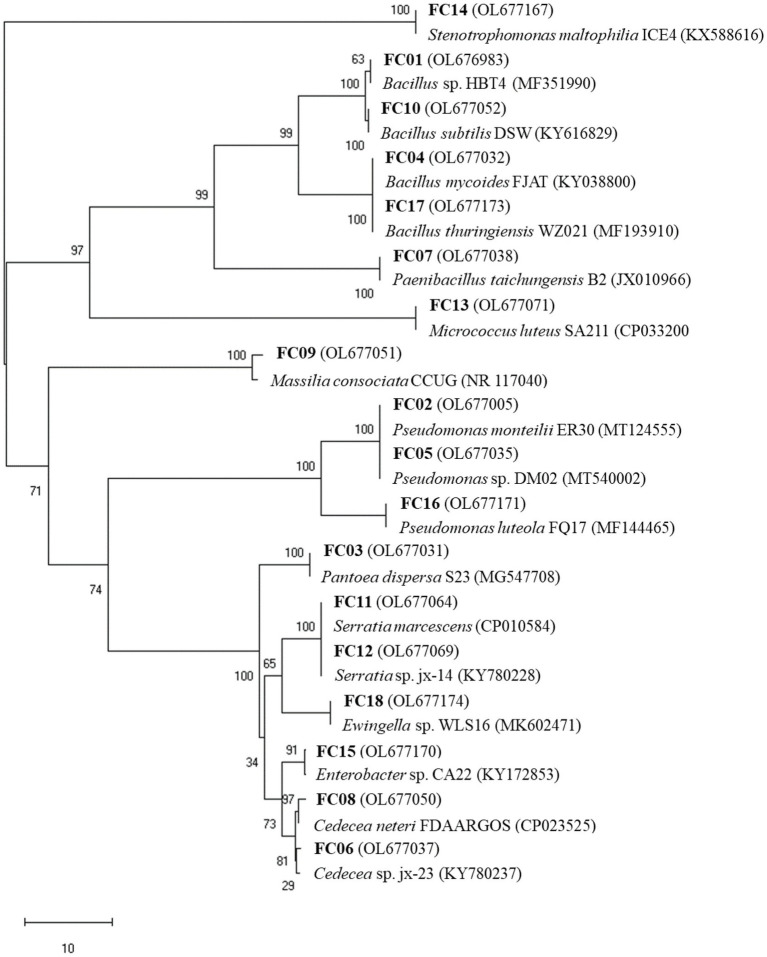
Phylogenetic tree of the 16S rRNA gene sequences of isolates based on the neighbor-joining method. Bootstrap values (1,000 replications) are indicated at tree branching points.

A total of 18 morphologically different bacterial colonies were isolated. All 16S rRNA gene sequences showed high similarities (≥99%) with sequences obtained from the NCBI database (Table 2). The 16S rRNA gene sequences of the 18 bacterial isolates were analyzed, and the phylogenetic tree was constructed (Figure 1). The sequence analyses of the 16S rRNA gene showed that the main isolated bacteria were related to the genera *Bacillus* (4 isolates), *Pseudomonas* (3 isolates), *Cedecea* (2 isolates), and *Serratia* (2 isolates; Table 2; Figure 1).

**Table 2:** Identification of the isolates based on the 16S rRNA gene sequence.

Isolates	GenBank accession number	GenBank closest match (accession number)	Sequence identity %
FC01	OL676983	*Bacillus* sp. HBT4 (MF351990)	100%
FC02	OL677005	*Pseudomonas monteilii* ER30 (MT124555)	100%
FC03	OL677031	*Pantoea dispersa* S23 (MG547708)	99%
FC04	OL677032	*Bacillus mycoides* FJAT (KY038800)	100%
FC05	OL677035	*Pseudomonas* sp. DM02 (MT540002)	100%
FC06	OL677037	*Cedecea* sp. jx-23 (KY780237)	99%
FC07	OL677038	*Paenibacillus taichungensis* B2 (JX010966)	99%
FC08	OL677050	*Cedecea neteri* FDAARGOS (CP023525)	99%
FC09	OL677051	*Massilia consociata* CCUG (NR 117040)	99%
FC10	OL677052	*Bacillus subtilis* DSW (KY616829)	100%
FC11	OL677064	*Serratia marcescens* (CP010584)	99%
FC12	OL677069	*Serratia* sp. jx-14 (KY780228)	99%
FC13	OL677071	*Micrococcus luteus* SA211 (CP033200)	100%
FC14	OL677167	*Stenotrophomonas maltophilia* ICE4 (KX588616)	100%
FC15	OL677170	*Enterobacter* sp. CA22 (KY172853)	99%
FC16	OL677171	*Pseudomonas luteola* FQ17 (MF144465)	99%
FC17	OL677173	*Bacillus thuringiensis* WZ021 (MF193910)	100%
FC18	OL677174	*Ewingella* sp. WLS16 (MK602471)	100%

### Effect of the Inoculation of Isolates on the Growth of *Lemna aequinoctialis*

**Figure 2:**
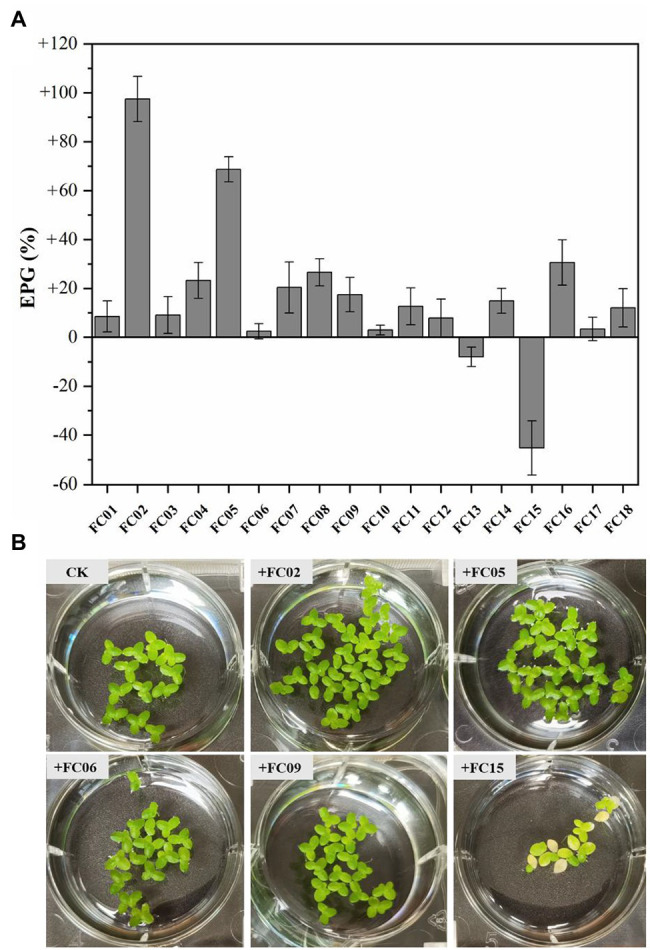
Effects on plant growth (EPG) of single isolated bacteria on plant growth **(A)**. EPG was evaluated by the change in fresh weight of *Lemna aequinoctialis* relative to that of aseptic control. Error bars show the standard errors (*n* = 3). Photograph images of duckweed inoculated with several strains after 7 days **(B)**.

A total of 18 isolates were examined for their effects on duckweed growth by inoculation with sterilized *L. aequinoctialis* in Hoagland solution. As shown in Figure 2, duckweed growth was affected both positively and negatively by the inoculation of isolates. One bacterial strain, namely FC02, showed the greatest plant growth-promoting activity compared to other isolates (Figure 2). The FC02 strain increased the plant biomass up to 1.97-fold compared with the corresponding uninoculated control. Similarly, *Pseudomonas* sp. Ps6 (Yamakawa et al., 2018) exhibited exceptional activity to promote *Lemna minor* growth by 2 ~ 2.5-fold in 10 days compared with aseptic plants. The growth-promoting activity of a previous isolate, *A. calcoaceticus* P23 (Yamaga et al., 2010), when tested under the same conditions, was 1.5–2-fold that of aseptic *L. minor*. Finally, strain FC02 with the greatest plant growth-promoting potential was selected for NEO removal experiments. To our knowledge, this is the first report of bacterial strain belonging to *P. monteilii* with the ability to promote the growth of duckweed.

### The Morphology and Plant Growth-Promoting Traits of the Selected Isolate FC02

**Figure 3:**
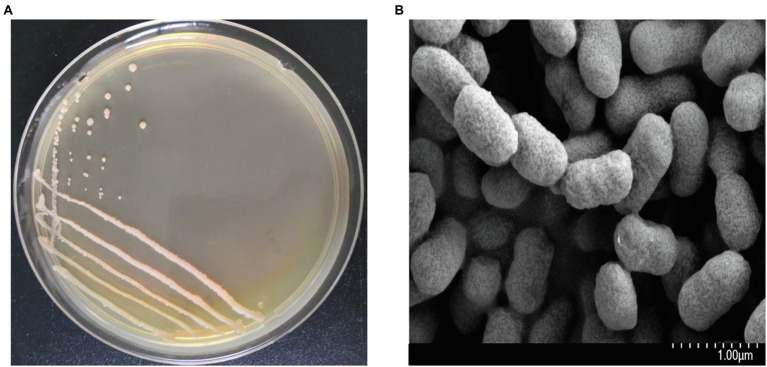
The colonial morphology **(A)** and scanning electron micrograph **(B)** of strain FC02.

The colonial and cell morphology of strain FC02 are shown in Figure 3. The plant growth-promoting properties of strain FC02 are presented in Table 3. Strain FC02 was found to be a producer of IAA, siderophores, and ACC-deaminase and to have the ability to dissolve potassium from insoluble P-bearing minerals, but it could not fix nitrogen. IAA is a crucial phytohormone that regulates plant development and growth (Chauhan et al., 2015). Idris et al. (2007) used different mutants of the PGPB *Bacillus amyloliquefaciens* with impaired IAA synthesis that was correlated with reduction in growth promotion of *L. minor*, revealing that IAA is a growth-promoting factor for duckweed plants. However, another study showed that the external addition of IAA did not significantly affect the growth of *L. minor* at all concentrations tested (Utami et al., 2018). Therefore, further investigation of the plant growth-promoting mechanisms of strain FC02 is necessary.

**Table 3:** Beneficial traits of *Pseudomonas monteilii* FC02.

Item	Results
IAA production (μg ml^−1^)	14.9 ± 0.7
Siderophore	+
Phosphate solubilization	+
ACC-deaminase	+
N_2_-fixation	−

“+” represents a positive reaction; “−” represents a negative reaction.

### Effects of NEOs on *Lemna aequinoctialis* Growth

**Figure 4:**
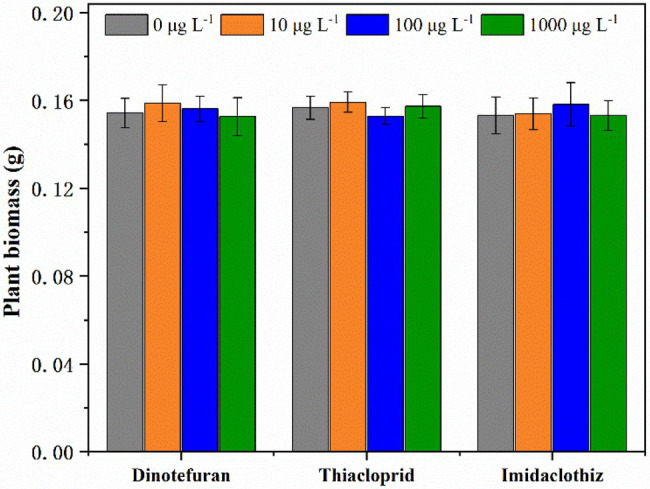
Plant biomass of *Lemna aequinoctialis* in toxicity experiments with NEOs. Error bars indicate the standard deviations (*n* = 3).

Τhe toxicity of target NEO compounds on *L. aequinoctialis* was tested at three different concentrations (10, 100, and 1,000 μg L^−1^). According to the results of duckweed toxicity experiments, for all tested concentrations, no significant differences (*p* < 0.05) were observed in *L. aequinoctialis* growth (Figure 4). To our knowledge, there are limited data available in the current literature on the toxicity of NEOs on *L. aequinoctialis*. Specifically, Anderson et al. (2015) reported that duckweed *Lemna gibba* and alga *Selenastrum carpricornutum* were tolerant to neonicotinoid clothianidin at very high concentrations (>100,000 μg L^−1^).

### Plant Biomass in the NEO Removal Experiment

Plant tolerance and growth in the presence of contaminants are of critical importance, as they can limit the efficiency of phytoremediation (Gatidou et al., 2017; Singh et al., 2019). In this study, the presence of NEOs in the medium did not significantly affect the growth of duckweed plants (*p* > 0.05) at a concentration of 100 μg L^−1^ (Table 4). The plant biomass results indicated that strain FC02 promoted duckweed plant growth in all three NEO-contaminated media (Table 4). In an earlier study, Yamaga et al. (2010) also showed that inoculation with *A. calcoaceticus* P23 significantly increased duckweed plant growth in phenol-contaminated medium.

**Table 4:** Plant biomass of inoculated and uninoculated duckweed in the NEO removal experiment.

Time (d)	DW− (g/beaker)			DW+ (g/beaker)		
	Dinotefuran	Thiacloprid	Imidaclothiz	Dinotefuran	Thiacloprid	Imidaclothiz
0	0.30 ± 0.00a	0.30 ± 0.00a	0.30 ± 0.00a	0.30 ± 0.00a	0.30 ± 0.00a	0.30 ± 0.00a
3	0.73 ± 0.07a	0.81 ± 0.04a	0.77 ± 0.03a	1.08 ± 0.09b	1.15 ± 0.11b	1.02 ± 0.07b
7	2.35 ± 0.12a	2.41 ± 0.18a	2.47 ± 0.26a	3.71 ± 0.19b	3.64 ± 0.12b	3.84 ± 0.10b
14	4.74 ± 0.13a	4.63 ± 0.16a	4.58 ± 0.24a	6.01 ± 0.23b	6.26 ± 0.31b	6.13 ± 0.15b

Treatments: DW−, uninoculated duckweed; DW+, duckweed inoculated with FC02. Values are mean ± standard deviations (*n* = 3). Different letters indicate significant differences (*p* < 0.05; Tukey’s test) among treatments.

### Persistence of Strain FC02 in the NEO Removal Experiment

The colonization and persistence of inoculated bacteria are crucial for their effectiveness in the phytoremediation process. The survival of inoculated bacteria was recorded in the plant tissues (DW+ treatment) and in the treated medium (FC02 treatment; Table 5). FC02 displayed high colonization capacity in the duckweed plant tissues throughout the 14-day experiment. After 7 days, the cell counts in duckweed plant tissues were up to 7.65, 7.28, and 7.80 × 10^6^ CFU g^−1^ in dinotefuran-, thiacloprid-, and imidaclothiz-contaminated medium, respectively. The high persistence of inoculated strain FC02 might be due to the interaction with plants, which is a source of nutrients and provides space for bacteria to attach and proliferate. On the other hand, in the unvegetated FC02 treatment, the cell counts in all three NEO-contaminated media generally tended to decrease during the whole experiment. This may be due to the lack of interaction between the two partners, which resulted in a decline in strain FC02 cells.

**Table 5:** Persistence of inoculated bacteria in the FC02 and DW+ treatments.

Time (d)	FC02 (×10^4^ CFU ml^−1^)			DW+ (×10^6^ CFU g^−1^ fresh weight)		
	Dinotefuran	Thiacloprid	Imidaclothiz	Dinotefuran	Thiacloprid	Imidaclothiz
0	2.06 ± 0.09	2.11 ± 0.14	2.17 ± 0.11	6.83 ± 0.12	6.87 ± 0.06	6.75 ± 0.26
3	1.87 ± 0.08	1.75 ± 0.21	1.91 ± 0.14	6.12 ± 0.25	6.43 ± 0.18	6.36 ± 0.31
7	1.17 ± 0.13	1.08 ± 0.09	1.21 ± 0.16	7.65 ± 0.16	7.28 ± 0.33	7.80 ± 0.29
14	0.45 ± 0.03	0.51 ± 0.08	0.57 ± 0.07	5.97 ± 0.36	5.62 ± 0.25	5.73 ± 0.20

Treatments: FC02, strain FC02 alone; DW+, duckweed inoculated with stain FC02. Values are mean ± standard deviations (*n* = 3).

### Removal of NEOs by Strain FC02 and *Lemna aequinoctialis* in Water

**Figure 5:**
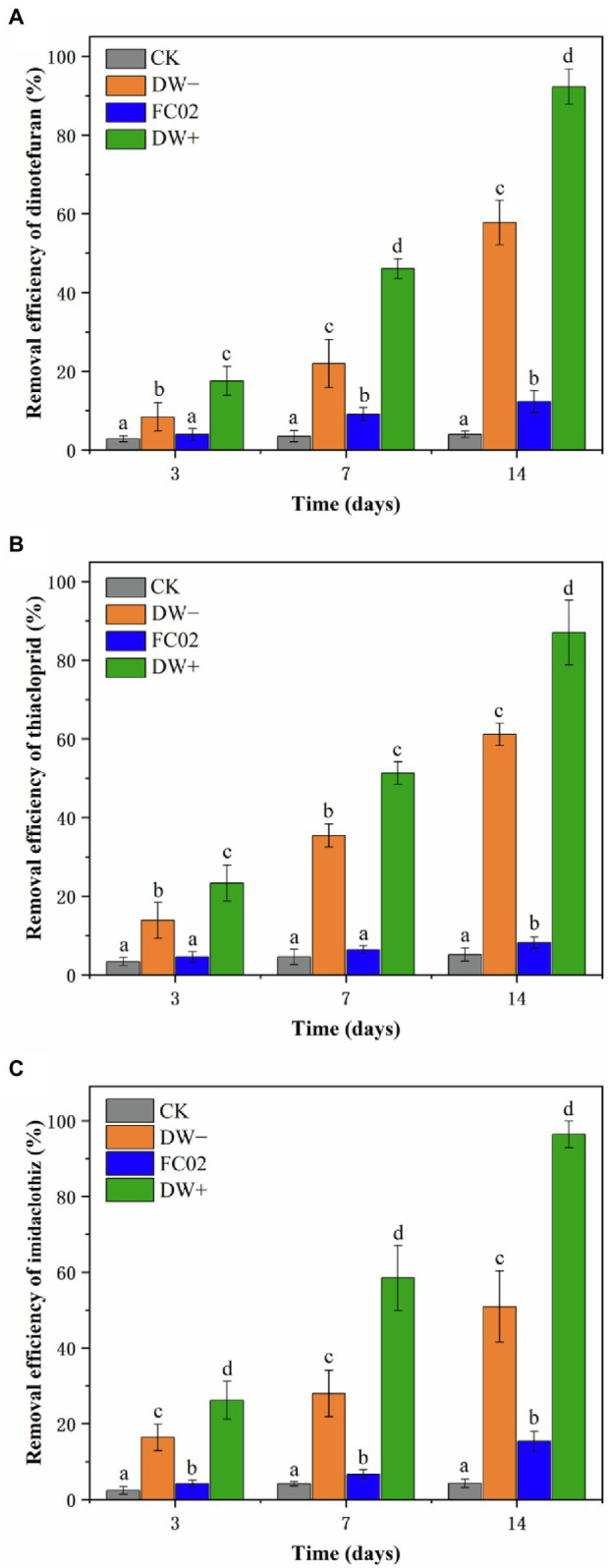
Removal efficiencies of dinotefuran **(A)**, thiacloprid **(B)**, and imidaclothiz **(C)** by duckweed and strain FC02. Treatments: CK, no duckweed and FC02; DW−, uninoculated duckweed; FC02, strain FC02 alone; DW+, duckweed inoculated with stain FC02. Error bars indicate the standard deviations (*n* = 3). Different letters indicate significant differences at *p* < 0.05 by Tukey’s test.

The efficiency of strain FC02, duckweed, and their combination for the removal of dinotefuran, thiacloprid, and imidaclothiz in water was studied. The results of the control group suggested that dinotefuran, thiacloprid, and imidaclothiz were persistent in water, with only 3.97, 5.13, and 4.28% eliminated at the end of the 14-day experimental period, respectively (Figure 5). Earlier studies also reported that abiotic loss of NEOs was negligible over the course of more than 7 days (Muerdter and LeFevre, 2019; Zhan et al., 2021). The concentrations of NEOs in the DW−, FC02, and DW+ treatments showed a decrease with time, and the maximum NEO removal was observed in the DW+ treatment, followed by that in the DW− and FC02 treatments. To our knowledge, this is the first study to demonstrate the ability of PGPB to enhance the phytoremediation efficiency of duckweed in NEO-contaminated water.

Microbial degradation, which is one of the important metabolic pathways of pesticides in the environment, is a simple and effective method for remediation of pesticide contamination (Myresiotis et al., 2015; Dai et al., 2021). Degradation of NEOs by microbes has been observed. For instance, *Pseudomonas* sp. 1G isolated from soil with a history of repeated exposure to pesticides decreased approximately 70% of 50 mg L^−1^ imidacloprid and thiamethoxam after 14 days, respectively (Pandey et al., 2009). Dai et al. (2021) reported that Actinomycetes *Rhodococcus ruber* is capable of biodegradation of the neonicotinoid insecticide nitenpyram *via* the hydroxylation pathway. Additionally, Zhao et al. (2019) have described the isolation of *Microvirga flocculans* CGMCC 1.16731, which is capable of two-step transformation of thiacloprid to 4-hydroxy thiacloprid *via* hydrolysis and hydroxylation. In this study, PGPB strain FC02 alone showed less degradation ability, accounting for the elimination of 12.35, 8.82, and 15.09% of dinotefuran, thiacloprid, and imidaclothiz, respectively, after 14 days (Figure 5). Further studies are needed to explore the degradation pathways and metabolites of these three pesticides.

The use of *L. aequinoctialis* significantly enhanced the removal of all three NEOs, indicating the critical role of plant uptake in their removal. Specifically, removal equal to 57.61, 61.38, and 50.91% was observed for dinotefuran, thiacloprid, and imidaclothiz, respectively, at the end of the 14-day exposure (Figure 5). Many studies have demonstrated the potential of duckweed for the remediation of several organic contaminants (Ekperusi et al., 2019; Panfili et al., 2019). For instance, *L. minor* was capable of removing 100% of benzotriazole after 10 days at an initial concentration of 150 μg L^−1^ (Gatidou et al., 2017). More than 60% removal of terbuthylazine (initial concentration of 250 μg L^−1^) was achieved after 14 days using *L. minor* (Panfili et al., 2019). A study by Singh et al. (2019) reported that the duckweed *Spirodela polyrhiza* efficiently removed 93.7% ofloxacin after 7 days at an initial concentration of 1,000 μg L^−1^. These studies are in agreement with our finding that duckweed can be a potential organism for the significant removal of pollutants from water. Little is known about the complete mineralization and transformation of NEOs in duckweeds (Ekperusi et al., 2019; Kafle et al., 2022). In this study, however, we did not detect any of the potential metabolites in *L. aequinoctialis*. Previous study showed that only two thiacloprid metabolites (thiacloprid amide, 6-chloronicotinic acid) were detected in the tissues of *Lemna turionifera* (Muerdter and LeFevre, 2019).

In the current investigation, enhanced removal of dinotefuran (92.23%), thiacloprid (87.75%), and imidaclothiz (96.42%) was observed when strain FC02 was combined with duckweed (Figure 5). Plant–bacterial association seems to be an effective approach for the removal of hazardous xenobiotics, including pesticides (Backer et al., 2018). For instance, the removal of three aromatic compounds (phenol, aniline, and 2,4-dichlorophenol) was obviously facilitated in the presence of root-associated bacteria (Toyama et al., 2006). Rhizosphere-associated bacteria of *Eichhornia crassipes* enhanced the removal of chlorpyrifos (Anudechakul et al., 2015). Continuous removal of phenol can be attributed to the beneficial symbiotic interaction between duckweed (*Lemna aoukikusa*) and *A. calcoaceticus* P23 (Yamaga et al., 2010). There must be some interaction between strain FC02 and duckweed plants. The plant root zone provides a good living environment for microbes, and plant exudates that are rich in sugars, carbohydrates, and amino acids increase microbial activity and promote the microbial biodegradation or metabolism of pollutants (Xun et al., 2015; Li et al., 2021; Zhan et al., 2021). Correspondingly, PGPB promotes plant growth and enhances the total root surface area, therefore increasing pesticide uptake by plant roots (Myresiotis et al., 2014; Li et al., 2021). This synergistic relationship enhances the role of each partner in pollutant removal (Anudechakul et al., 2015; Yan et al., 2021). Some previous studies showed a correlation between biomass increase and pollutant degradation with the help of some bacteria (Myresiotis et al., 2014; Zhan et al., 2021). In our study, significant differences in plant growth were observed between inoculated and uninoculated duckweed plants (Table 4), which might indicate the direct contribution of biomass growth to the degradation of NEOs in duckweed. In addition, considering that co-metabolism is the most common mechanism used by organic pollutant-degrading bacteria (Bhanse et al., 2022), it would be possible that strain FC02 would have used duckweed root exudates as energy source to degrade NEOs. Alvarez et al. (2022) also reported that biodegradation of hexachlorocyclohexane isomers by *Sphingobium* sp. D4 was enhanced in the presence of maize root exudates. Furthermore, Daudzai et al. (2018) observed that the inoculation of *Clitoria ternatea* with PGPB *Bacillus cereus* significantly increased the expression of plant ethylbenzene degradation genes and improved ethylbenzene removal efficiency.

### NEO Concentrations in *Lemna aequinoctialis*

**Figure 6:**
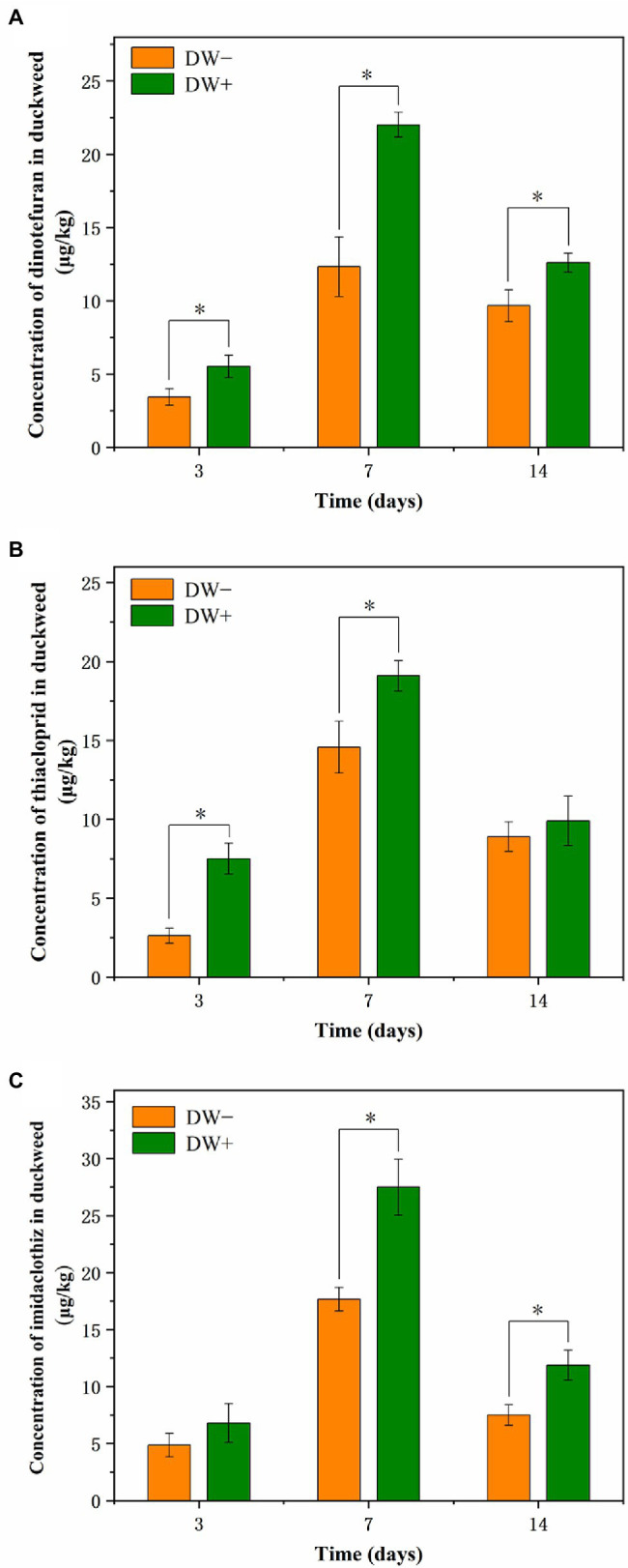
Concentrations of dinotefuran **(A)**, thiacloprid **(B)**, and imidaclothiz **(C)** in the inoculated and uninoculated duckweed plants. Treatments: DW−, uninoculated duckweed; DW+, duckweed inoculated with stain FC02. Error bars represent standard deviations (*n* = 3). Asterisks indicate a significant difference between treatments on the same day (*p* < 0.05, *t*-test).

All three NEOs were detected in the tissues of *L. aequinoctialis* (including uninoculated and inoculated *L. aequinoctialis*) during the 14-day experimental period (Figure 6). As displayed in Figure 6, the concentrations of the pesticides in both uninoculated and inoculated *L. aequinoctialis* increased between Day 3 and Day 7 and then decreased thereafter. In particular, the inoculation of strain FC02 led to an increase in the concentrations of three NEOs in plant tissues in relation to the uninoculated *L. aequinoctialis* during the entire experiment. Similarly, Myresiotis et al. (2015) found that the concentrations of thiamethoxam in PGPB *Bacillus subtilis* FZB2-treated corn plants (1.53 mg kg^−1^) were significantly higher (*p* < 0.05) than the 0.62 mg kg^−1^ in the untreated control plants. In addition, it has been reported that the inoculation of plants with specific PGPR strains results in enhanced uptake of acibenzolar-S-methyl in tomato plants (Myresiotis et al., 2014). The enhanced pesticide residues in plant tissues may be due to several processes that take place between plants and PGPR (Rani et al., 2019). It has been reported that PGPR strains promote root growth and enhance root surface area, therefore increasing its absorption capacity by plant roots, which may explain the increased uptake of NEOs (Li et al., 2021; Yan et al., 2021). Once taken up by the roots, organic pollutants can be translocated to other tissues of the plant, such as stem and leaf (Kafle et al., 2022). Böttcher and Schroll (2007) found that most of the herbicide isoproturon taken up by duckweed *L. minor* accumulated in the fronds. In this study, we detected the NEO concentrations in the whole plant of duckweed, which is consistent with previous studies (Gatidou et al., 2017; Muerdter and LeFevre, 2019). In plant cells, organic pollutants could be degraded *via* metabolic processes (Ekperusi et al., 2019). Possible biochemical reactions include the transformation of parent chemicals to nonphytotoxic metabolites, the conjugation of metabolites with macromolecules, and the incorporation of these conjugated products into plant vacuoles and cell walls (Yamaga et al., 2010; Ekperusi et al., 2019; Zhan et al., 2021). Additionally, extracellular processes may also be important in the duckweed-mediated removal of organic pollutants from solution (Ziegler et al., 2016). For example, Reis et al. (2014) found that phenolic endocrine-disrupting chemicals could be oxidative degraded by duckweed cell wall-bound peroxidases.

## Conclusion

To increase the efficiency of NEO elimination in water, a novel plant growth-promoting bacterium, FC02, identified as *P. monteilii*, was isolated and used in combination with duckweed. The inoculation of PGPB-stimulated plant biomass production and the uptake of NEOs compared with the uninoculated plants. The removal efficiency of dinotefuran, thiacloprid, and imidaclothiz in the inoculated duckweed treatment was greater than that of the addition of PGPB and the duckweed plant alone. The possible mechanisms resulting in the improved duckweed phytoremediation efficiency were: (i) inoculation of strain FC02 increased the plant biomass *via* biosynthesis of the phytohormones, and (ii) duckweed enhanced the growth and biodegradation capacity of the adhered strain FC02. Overall, these results strongly suggest that the PGPB–duckweed partnership might be an effective and ecological alternative to accelerate the removal of NEOs present in water. However, further studies are needed to reveal the molecular mechanisms of duckweed–FC02 interactions and the metabolic pathways of NEOs in strain FC02 and duckweed.

## Data Availability Statement

The datasets presented in this study can be found in online repositories. The names of the repository/repositories and accession number(s) can be found in the article/supplementary material.

## Author Contributions

HW-W designed the research, analyzed the data, and wrote the article. XY-C, MX, Y-XZ, and YS performed the research. All authors contributed to the article and approved the submitted version.

## Funding

This work was supported by the National Natural Science Foundation of China (No. 31800103).

## Conflict of Interest

The authors declare that the research was conducted in the absence of any commercial or financial relationships that could be construed as a potential conflict of interest.

## Publisher’s Note

All claims expressed in this article are solely those of the authors and do not necessarily represent those of their affiliated organizations, or those of the publisher, the editors and the reviewers. Any product that may be evaluated in this article, or claim that may be made by its manufacturer, is not guaranteed or endorsed by the publisher.

## Footnotes

^1^ http://nabrc.org.cn/#/subResource/cccap
